# Bandwidth Enhancement and Frequency Scanning Array Antenna Using Novel UWB Filter Integration Technique for OFDM UWB Radar Applications in Wireless Vital Signs Monitoring

**DOI:** 10.3390/s18093155

**Published:** 2018-09-19

**Authors:** MuhibUr Rahman, Mahdi NaghshvarianJahromi, Seyed Sajad Mirjavadi, Abdel Magid Hamouda

**Affiliations:** 1 Department of Electrical Engineering, Polytechnique Montreal, Montreal, QC H3T1J4, Canada; muhibur.rahman@polymtl.ca; 2 Department of Electrical and Computer Engineering, McMaster University, Hamilton, ON L8S4L8, Canada; 3 Health Technology Incubator, Jahrom University of Medical Sciences, 74148-46199 Jahrom, Iran; 4 Department of Mechanical and Industrial Engineering, College of Engineering, Qatar University, Doha 2713, Qatar; seyedsajadmirjavadi@gmail.com (S.S.M.); hamouda@qu.edu.qa (A.M.H.)

**Keywords:** frequency scanning fan beam array antenna, wide-band applications, miniaturized band-pass filter, DGS (Defective Ground Structures), grounded coplanar waveguide (GCPW), grounded reflector, SCSRR (Semi-Complementary Split Ring Resonator)

## Abstract

This paper presents the bandwidth enhancement and frequency scanning for fan beam array antenna utilizing novel technique of band-pass filter integration for wireless vital signs monitoring and vehicle navigation sensors. First, a fan beam array antenna comprising of a grounded coplanar waveguide (GCPW) radiating element, CPW fed line, and the grounded reflector is introduced which operate at a frequency band of 3.30 GHz and 3.50 GHz for WiMAX (World-wide Interoperability for Microwave Access) applications. An advantageous beam pattern is generated by the combination of a CPW feed network, non-parasitic grounded reflector, and non-planar GCPW array monopole antenna. Secondly, a miniaturized wide-band bandpass filter is developed using SCSRR (Semi-Complementary Split Ring Resonator) and DGS (Defective Ground Structures) operating at 3–8 GHz frequency band. Finally, the designed filter is integrated within the frequency scanning beam array antenna in a novel way to increase the impedance bandwidth as well as frequency scanning. The new frequency beam array antenna with integrated band-pass filter operate at 2.8 GHz to 6 GHz with a wide frequency scanning from the 50 to 125-degree range.

## 1. Introduction

The Federal Communication Commission have assigned 3.1 GHZ to 10.6 GHz frequency band for Ultra-Wide Band (UWB) wireless communication [1]. Within this technology, two subsets of UWB exist, termed as (i) UWB orthogonal frequency division multiple access (OFDM-UWB) having frequency band of (3.43–4.48 GHz) and (6.60–10.2 GHz), (ii) Direct sequence UWB (DS-UWB) (3.1–4.85 GHz) and (6.20–9.70 GHz) [2].

In this regard, different array antennas with sub-radiators and extended reflector for different applications have been developed in the past decade [3,4,5,6,7,8]. The non-parasitic reflector is first presented in References [8,9]. In Reference [8], they simulated a fan beam array antenna to operate in Ku band while in [9] they developed a millimeter wave antenna having lightweight for the 60 GHz frequency band. These antennas achieve higher gain having more than 13 dBi, however, they suffer from narrower input impedance. Therefore, it is necessary to develop a new fan beam array antenna with wider impedance bandwidth performance. In References [10,11], the authors reported fractal antennas, for broadening impedance bandwidth using CPW feeding technique. In Reference [11], the authors applied the GCPW concept on the fractal antenna to achieve a wider bandwidth.

Also, designing a frequency scanning antenna is a hot topic and many frequency scanning antennas have been developed in the literature [12,13,14]. Recently, in Reference [15] they reported an antenna for frequency scanning by utilizing a strip-line as an intended transmission line for the coupler structure. This antenna can be shifted to different angles based on the coupler structure which limits the antenna performance. Nevertheless, an antenna having the capability of both scanning and enhanced bandwidth is reported so far. Different bandstop filters integrated with microstrip antennas have been reported in References [16,17,18,19] for band-notching purposes, however, no integrated filter with fan beam array antenna has also been reported so far. This manuscript will introduce the concept of bandpass filter integration in array antennas for enhancing bandwidth performance.

The concept of bandwidth enhancement and antenna miniaturization using reactive impedance surface (RIS) has been introduced in Reference [20]. They designed different planar antennas such as patch and dipole on RIS and compared their characteristics with the same antennas over PMC and PEC. Similarly, in Reference [21] bandwidth and gain enhancement of Microstrip antenna is achieved using planar patterned metamaterial concept. Also, in Reference [22] wide bandwidth antenna as a passive antenna sensor is implemented and tested for temperature sensing without any electronics in the design.

In this paper, we first introduced a fan beam array antenna that has the capability to operate at the 3.3 GHz and 3.5 GHz frequency bands. This antenna basically comprises of three components having a CPW fed line, GCPW radiating element, and grounded reflector. It is displayed that the grounded reflector greatly minimizes the back lobe level. Secondly, a miniaturized wideband bandpass filter is designed and fabricated which operate from 3 GHz to 8 GHz frequency band. This filter is specially designed for integration purposes. Finally, the fan beam array antenna and a bandpass filter is integrated in a very novel way to enhance the bandwidth and increase frequency scanning. The technique of integration is based on the placement of the filter in CPW fed line, but opposite to the excitation side of the feedline. The filter is matched with the array antenna and operate from 2.8 GHz to 6 GHz frequency range. It is seen that a wide frequency scanning is achieved by placing the proposed filter at the CPW fed line. The equivalent circuit of the integrated bandpass filter as well as proposed antenna, is also provided for validation purpose. The comparison between the proposed antenna and some related designs in terms of designing technique, frequency scanning, and bandwidth enhancement is summarized in Table 1.

The arrangement of the manuscript is carried out in the following manner: Section 2 deals with the development of fan beam array antenna. Section 3 deals with the design guidelines of the developed fan beam antenna array. The measured and simulated results of the developed fan beam antenna array are shown in Section 4. The development of a miniaturized wideband bandpass filter with simulated and measured response is carried out in Section 5. The development of bandwidth-enhanced frequency scanning proposed fan beam array antenna is carried out in Section 6. Section 7 deals with measured and simulated and results of the proposed frequency scanning fan beam array antenna. Section 8 deals with the application of the proposed antenna in target detection, which is followed by the Conclusion.

## 2. Development of Fan Beam Array Antenna

**Figure 1:**
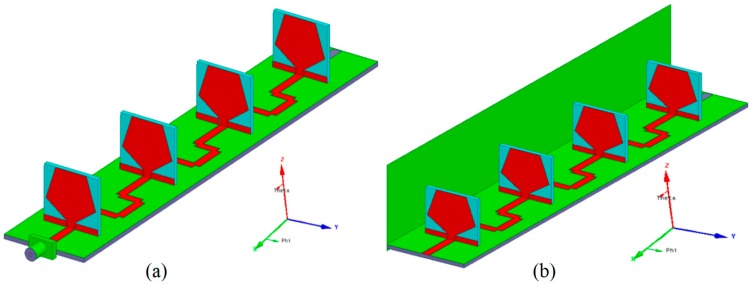
CPW-feed non-planar linear array (**a**) Fan beam antenna array without reflector (perspective view); (**b**) Fan beam antenna array with reflector (perspective view).

Three different concepts are combined to develop a fan beam antenna having a non-parasitic structured grounded-reflector, CPW feed network, and GCPW radiation element as shown in Figure 1. The developed linear fan beam array antenna is designed to operate at 3.5 GHz WiMAX application. Figure 1 display the perspective outlook of the fan beam antenna array with and without a reflector. The reflector and radiating element are perpendicularly linked to the CPW line as shown in Figure 1b.

The array factor for the far zone of the linear array is made in-line with the *X*-axis using the following equation [18,19,20,21,22,23,24]

(1) $$AF(\theta ,\phi )={\sum_{n=1}^{M}a_{n}e}^{jn(\frac{2\pi }{\lambda }d\sin\theta \cos\phi +\alpha )}$$

where, represents the angles b/w the intended axis of the designed array and observer radial vector with respect to the origin, *a_n_* is the excitation amplitude, *α* represents wave progression between array elements, *d* is the distance between any two array elements, and is the designed wavelength of the array elements, and *λ* is the designed wavelength of the array.

**Figure 2:**
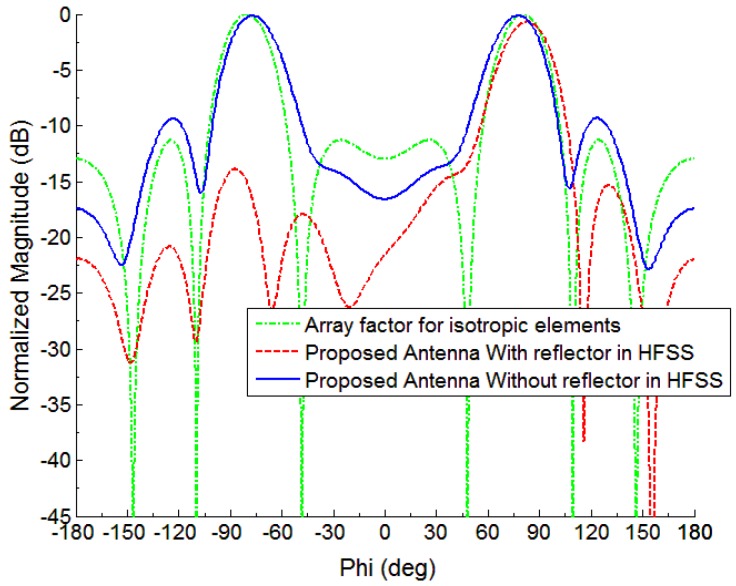
Radiation pattern at f = 3.5 GHz for a linear array having four elements in case of isotropic, actual monopole, and grounded reflector of the proposed frequency scanning fan beam array antenna.

The four elements linear aligned array is developed by selecting *d* = 0.5*λ* and α=−π/6. Figure 2 represents the array factor developed in the xy-plane with max. SLL (Side lobe level) of −11.31 dB with a 26.7 degree of beam width. Figure 2 also shows the simulated pattern of the monopole element array backed by the reflector and without a reflector. It is clear from Figure 1 that the grounded-reflector has greatly minimized the back lobe level, which is very advantageous.

Previously in Reference [23], a fan-beam antenna is realized utilizing six elements conventional planar monopole array antenna and feed network. Dolph-Tschebyscheff distribution is employed and a broadband array feed network is designed to satisfy beneficial input impedance bandwidth requirements in the frequency range 1.70–2.20 GHz [23]. However, all of these antennas are designed using planar radiation elements. In addition, planar monopole elements need a symmetrical reflector. Therefore, spatial dimensions can be reduced by conventional non-planar monopole antenna as a radiation elements, and this is mainly because of the image theory [24], so the reflector height is reduced to half. Even so, non-planar array antennas do not possess easy installation, lightweight, or cheap characteristics, mainly because of their feed network. In order to address these problems, we have combined a grounded coplanar waveguide (GCPW) radiating element with a CPW fed line, which is the best choice to address such feeding network problems.

## 3. Design Guidelines of Fan Beam Array Antenna

**Figure 3:**
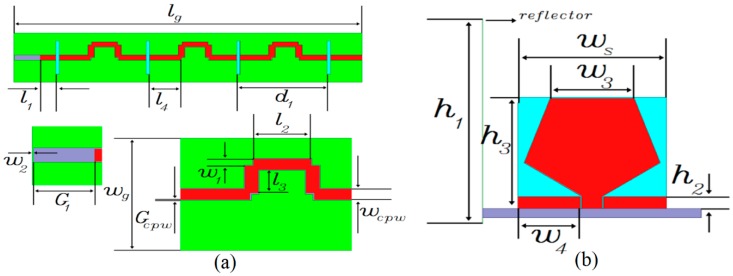
(**a**) CPW feed line; (**b**) Geometrical parameters of the developed single element of the fan beam array.

**Figure 4:**
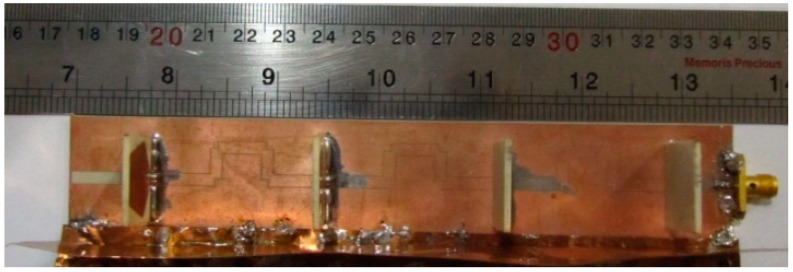
Fabricated fan beam array antenna.

The fan beam array antenna is developed and its CPW fed line is shown in Figure 3a while the radiating element with reflector is revealed in Figure 3b. The antenna is comprised of three main components having GCPW radiating element, CPW feed line, and grounded reflector. The CPW feed line and radiating element part is designed using Rogers RO4003 substrate with loss tangent of 0.0027 and dielectric constant of 3.38. The other parameters are: *d*_1_ = 42.86 mm, *G_cpw_* = 0.20 mm, *w_g_* = 30.0 mm, *l_g_* = 165.0 mm, *w_cpw_* = 2.80 mm, *G*_1_ = 12.25 mm, *l*_1_ = 7.40 mm, *w*_1_ = 1.80 mm, *l*_2_ = 11.93 mm, *w*_2_ = 0.25 mm, *l*_3_ = 6.22 mm, *w*_3_ = 11.52 mm, *l*_4_ = 14.83 mm, *w*_4_ = 8.5 mm, *w_s_* = 20.4 mm, *h*_3_ = 19.0 mm, *h*_2_ = 2.0 mm, and *h*_1_ = 35.0 mm. The fabricated frequency fan beam antenna array is shown in Figure 4.

## 4. Simulation and Measurements of the Developed Fan Beam Array Antenna

**Figure 5:**
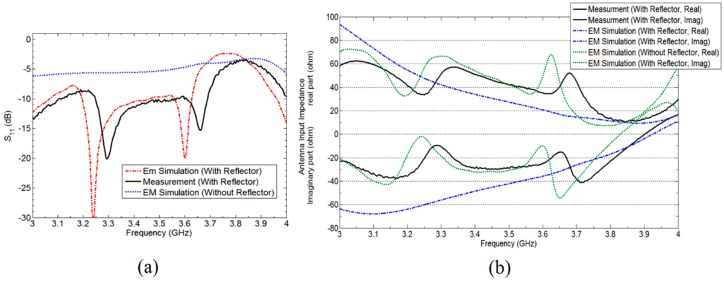
(**a**) S11 plot of the developed antenna array having fan beam with and without reflector; (**b**) Real and imaginary part of fan beam array antennas input impedance.

**Figure 6:**
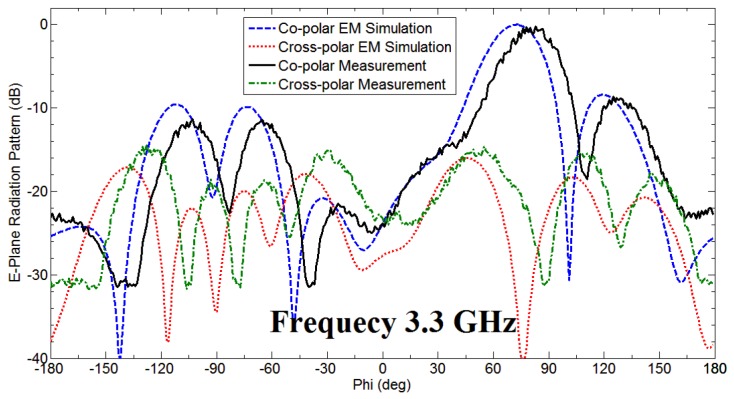
Normalized E-plane pattern of the developed antenna array having fan beam at 3.30 GHz.

**Figure 7:**
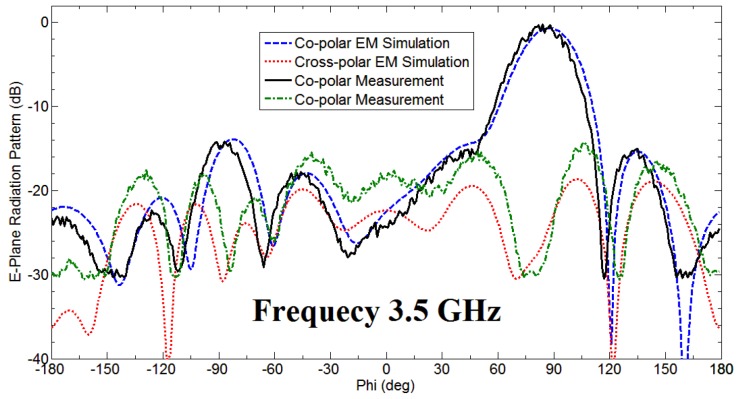
Normalized E-plane pattern of the developed antenna array having fan beam at 3.50 GHz.

**Figure 8:**
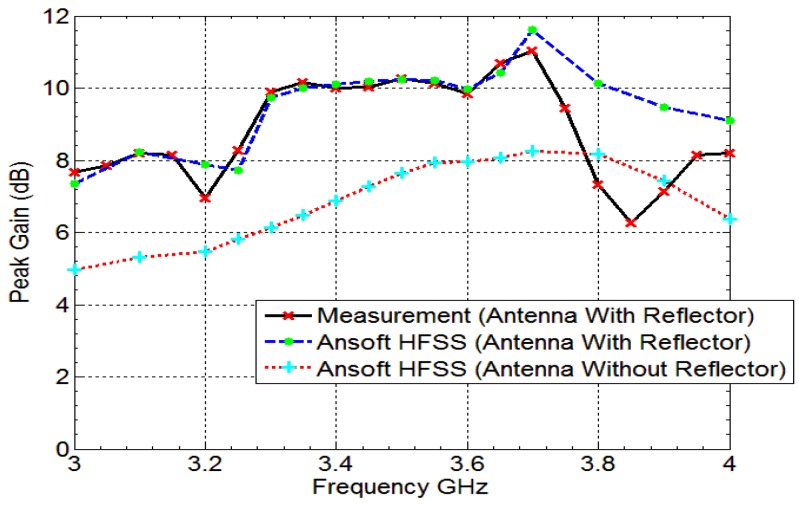
Measured and Simulated Gain (dB) of the developed antenna array having fan beam with and without a reflector.

The fan beam array antenna is designed and fabricated, and the response is measured as well. The simulated and measured S-parameter response is shown in Figure 5a. Also, Figure 6 and Figure 7 represents a normalized E-plane radiation pattern of the fan beam antenna array at 3.3 GHz and 3.5 GHz, respectively. Figure 6 and Figure 7 also shows that one side lobe is entirely merged with the main lobe due to the grounded reflector. It also shows that the back lobe level is considerably reduced by adding a grounded reflector as judged from Figure 2. Also, Figure 8 displays the simulated and measured antenna gain with and without a reflector. The overall performance of the antenna can be summarized well in Table 2.

**Figure 9:**
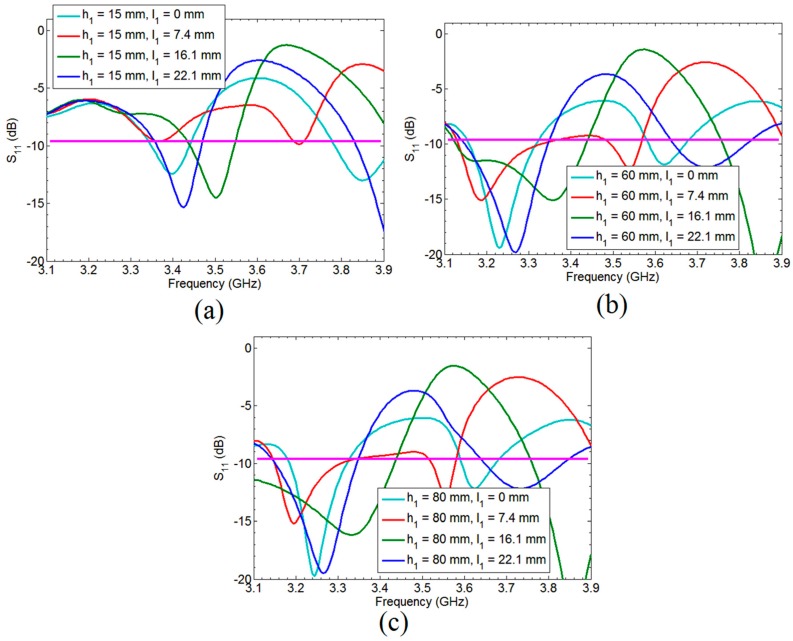
Parametric analysis of the Critical parameters of the fan beam array antenna; (**a**) Different *l*_1_ values having *h*_1_ = 15 mm; (**b**) Different *l*_1_ values having *h*_1_ = 60 mm; (**c**) Different l_1_ values having *h*_1_ = 80 mm.

Parametric analysis has been performed to show the critical parameters which affect the performance of the antenna. The height of the reflector *h*_1_ and distance between last array element and filter position *l*_1_ has been simulated for different values as shown in Figure 9. It can be seen from Figure 9a–c that the by changing *l*_1_ at same value of reflector height changes the antenna response. So proper combination of *h*_1_ and *l*_1_ is very important to achieve our desired response. Also, for planar type of antenna reflector should be symmetrical for the best performance, so the height of reflector is reduced here using the concept of image theory from 70.0 mm to 35.0 mm.

Figure 5a demonstrates S11 parameter magnitude (dB) (simulation results) employing Ansoft HFSS as well as the measurement results obtained for the proposed antenna and without reflector antenna. The measured input impedance bandwidth for VSWR less than 2 is 3250–3700 MHz, which is 12.94% fractional band-width for proposed antenna with grounded reflector. In addition, the Figure 5a shows that the grounded reflector improves the antenna reflection coefficient, noticeably. In addition, this effect was observed previously in Reference [23] when grounded reflector improved input impedance band width through 1.55–1.75 GHz in part of operating band. The concept of improving the input impedance by grounded reflector in early attempt to make use of the broad-banding properties of antennas is dealt with in our previous work of References [22,23]. The real and imaginary part of proposed antennas input impedance is shown in Figure 5b. This figure is a very clear example, which shows that a grounded reflector can improve input antenna impedance bandwidth as well as radiation pattern characteristics.

## 5. Development of Wideband Bandpass Filter with Simulated and Measured Results

**Figure 10:**
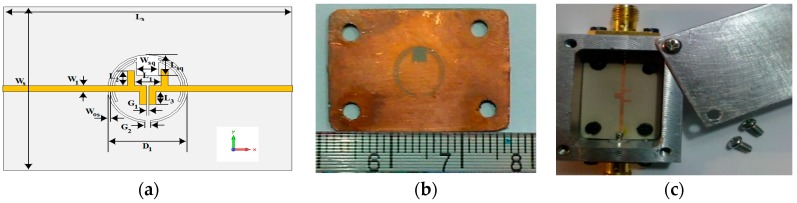
(**a**) Schematics and geometrical diagram of the SCSRR based bandpass filter; (**b**) Back-side of the fabricated bandpass filter; (**c**) Front-side of the fabricated bandpass filter.

**Figure 11:**
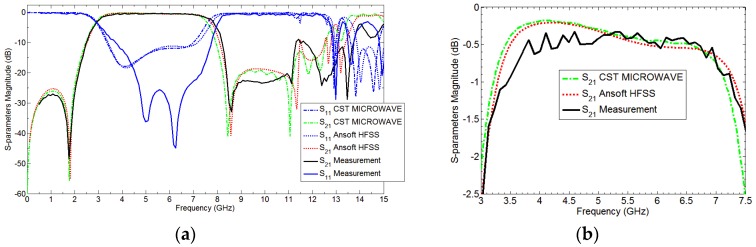
(**a**) Measured and Simulated frequency response of the SCSRR based miniaturized bandpass filter; (**b**) Zoom-in of part (**a**) from 3 GHz to 7.5 GHz.

The SCSRR based miniaturized wideband bandpass filter is designed and fabricated as shown in Figure 10. The dimensions of the substrate are: *Ws* × *Ls* = 17.6 × 26.0 mm^2^ while other parameters are: *L*_1_ = 2.4 mm, *W*_1_ = 0.6 mm, *L*_2_ = 1.55 mm, *G*_2_ = 0.55 mm, *L*_3_ = 1.4 mm, *G*_1_ = 0.25 mm, *W*_sq_ = 2.0 mm, *W*_os_ = 0.2 mm, *L*_sq_ = 2.0 mm, and *D*_1_ = 7.2 mm. This filter has the ability to operate at 3 GHz to 7 GHz frequency range with low isolation. The simulated and measured frequency response of the developed wideband bandpass filter is shown in Figure 11, which shows that the filter operates well from the 3 GHz to 7.5 GHz frequency range.

## 6. Development of Bandwidth-Enhanced Frequency Scanning Fan Beam Array Antenna

**Figure 12:**
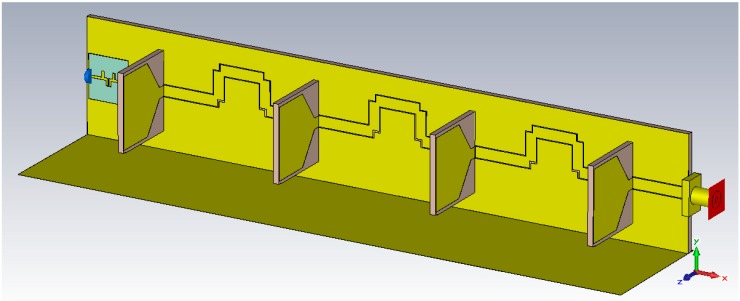
Perspective view of the Proposed Bandwidth-Enhanced Frequency Scanning Fan Beam Array Antenna with bandpass filter integrated into the feedline.

**Figure 13:**
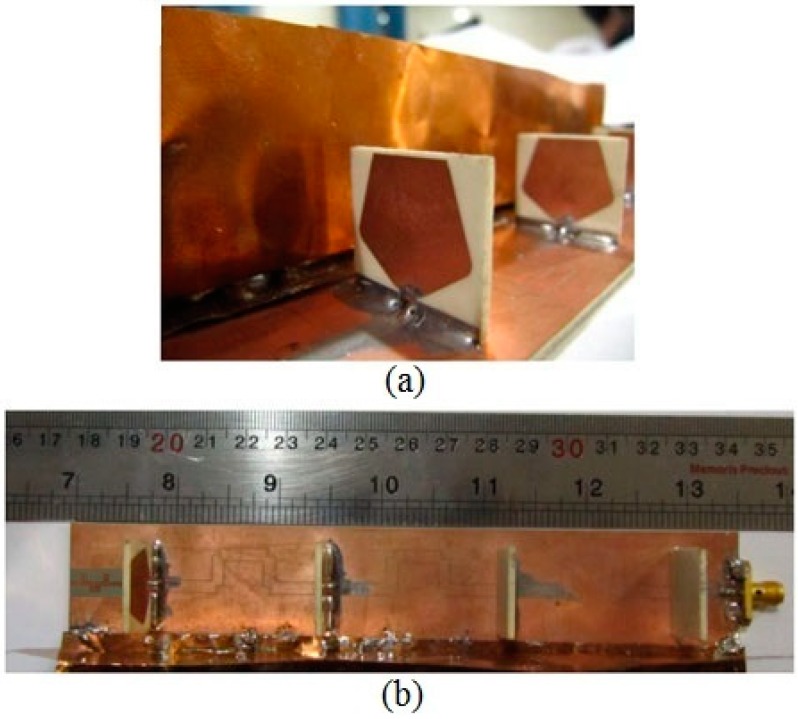
Fabricated Picture of the Bandwidth-Enhanced Frequency Scanning Fan Beam Array Antenna with bandpass filter integrated into the feedline (**a**) Perspective view; (**b**) Top view.

The Proposed bandwidth-enhanced frequency scanning fan beam array antenna is developed by integrating the miniaturized wideband bandpass filter with fan beam array antenna in their opposite side of the excitation. The schematic sketch of the proposed antenna with integrated filter is shown in Figure 12. As can be seen from the Figure 11 that the filter is placed in CPW fed line and matched for 2.8 GHz to 6 GHz frequency range. This technique is very promising and this type of filter integration within the antenna has not yet been reported in the literature. The proposed bandwidth-enhanced frequency scanning fan beam array antenna with integrated bandpass filter is also fabricated and is shown in Figure 13.

**Figure 14:**
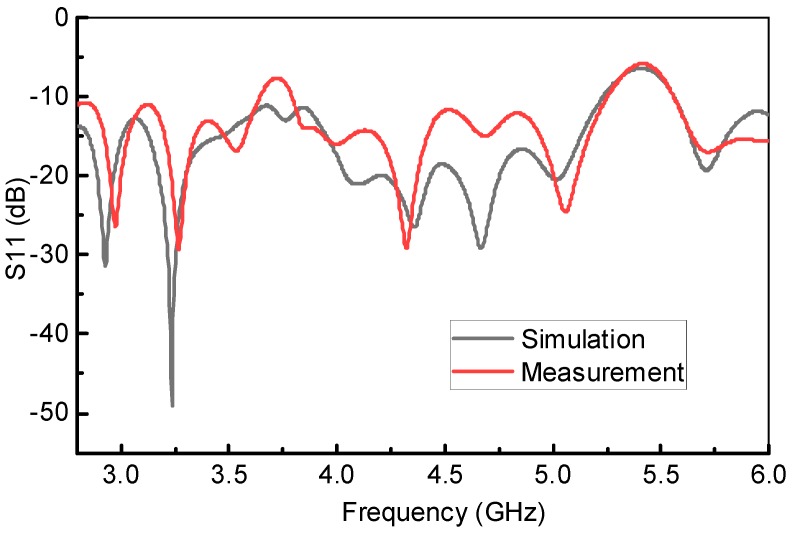
Simulated and Measured response of the Bandwidth-Enhanced Frequency Scanning Fan Beam Array Antenna with an integrated bandpass filter.

The response of the developed miniaturized bandpass filter is shown in Figure 11, which has a very promising frequency response and can be used as a UWB bandpass filter. The size of the filter is very small, and it is placed in the developed fan beam array antenna having a narrowband response. Different wideband bandpass resonators have been tested for achieving our desired response, but the proposed miniaturized filter provides the most promising response and improves matching of the antenna over a wide bandwidth as shown in Figure 14. Moreover, the proposed filter is very small and has been integrated within the antenna in order to maintain the circuit size.

## 7. Simulation and Measurement of the Proposed Antenna

**Figure 15:**
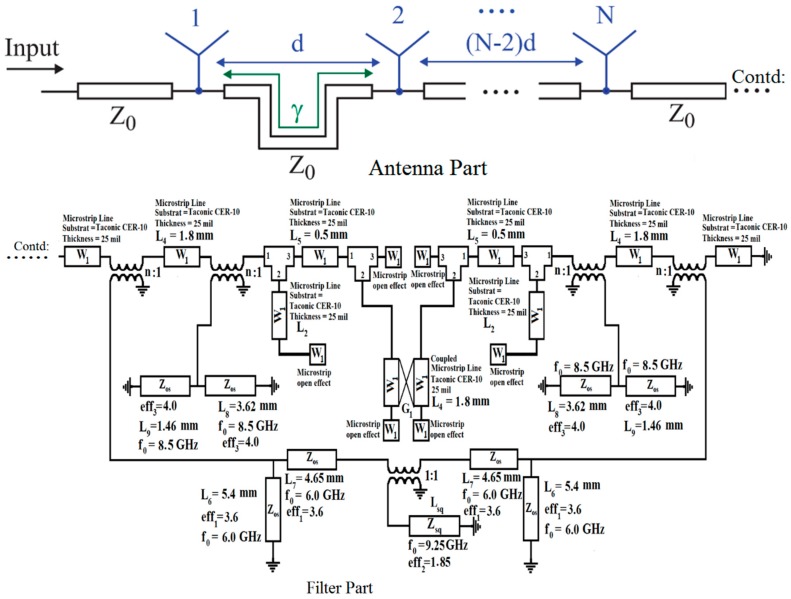
Equivalent circuit model developed for the filter and antenna.

**Figure 16:**
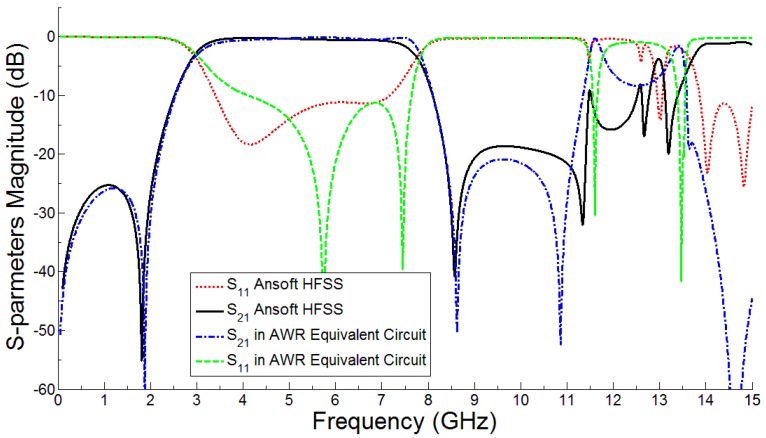
Frequency response comparison between HFSS and AWR for filter equivalent circuit validation.

The simulated and measured frequency response of the proposed bandwidth-enhanced frequency scanning fan beam array antenna integrated with miniaturized wideband bandpass filter is shown in Figure 14. It clearly shows that the bandwidth of the fan beam array antenna is considerably increased by placing the bandpass filter in the feedline. It can be seen from Figure 5 that the fan beam array antenna without integrated bandpass filter operate up to 3.8 GHz, while by integrating the filter, the bandwidth is enhanced and the proposed antenna operates from the 2.8 GHz to 6 GHz frequency range. The equivalent circuit model of the proposed bandwidth-enhanced frequency scanning fan beam array antenna integrated with miniaturized wideband bandpass filter is shown in Figure 15. Some required values for the equivalent circuit model are calculated and listed in Table 3. The equivalent circuit of the filter part is simulated in AWR and compared with the simulation from HFSS and it is seen that the equivalent circuit response is almost the same as shown in Figure 16.

**Figure 17:**
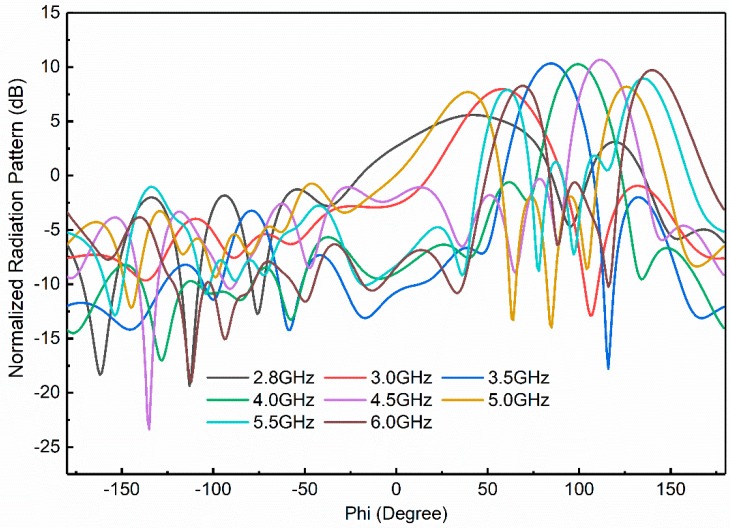
Normalized radiation pattern of the proposed developed frequency scanning fan beam array antenna with an integrated bandpass filter.

**Figure 18:**
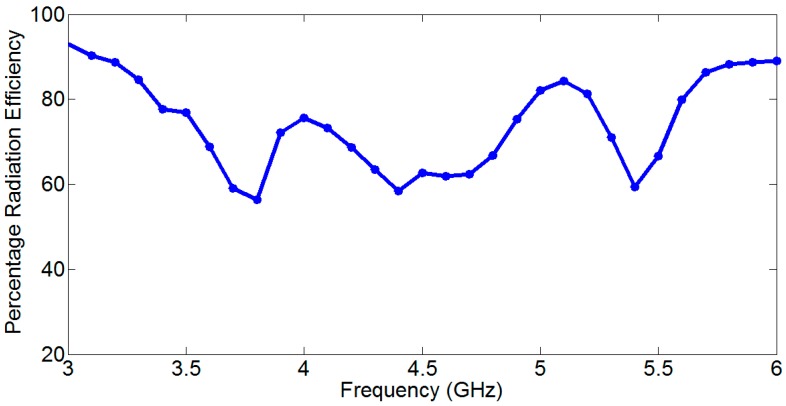
Percentage radiation efficiency of the proposed developed frequency scanning fan beam array antenna with an integrated bandpass filter.

The frequency scanning of the proposed antenna with integrated bandpass filter from 2.8 GHz to 6 GHz is displayed in Figure 17. It is clear that a wide frequency scanning is achieved from 50 to 125 °C due to the grounded reflector. The grounded reflector greatly reduces the back lobes as shown in Figure 16. It is seen that at 2.8 GHz the main beam is at 50 °C while at 6 GHz the main beam is shifted to almost 125 °C. This behavior makes the antenna additionally advantageous for use in OFDM-UWB communication applications. The percentage radiation efficiency of the proposed developed frequency scanning fan beam array antenna with an integrated bandpass filter is shown in Figure 18. The percentage radiation efficiency of the proposed antenna is within the range of approximately 60% to 92% within the passband. The radiation efficiency shows the bandpass filter effect for antenna input impedance match.

## 8. Application in Robotics for Target Detection

The proposed frequency scanning antenna can be used for target detection in Robotics. The scanning operation for target location with physical movement can be performed as single sided here. It is also achieved that if the proposed antenna is fed from two opposite ends at same frequency, two counter scanning beams can be achieved. Due to this property of the proposed scanning antenna, it can be turned around the physical center, and we can establish angle target detection mechanism easily. The object can be easily tracked from received power against the turn angle by null pointing.

**Figure 19:**
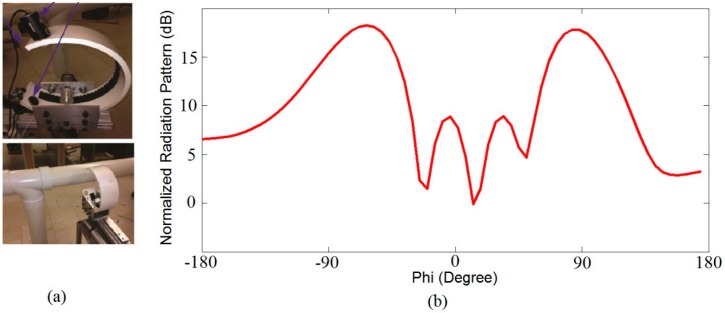
(**a**) C-arms of the robot where both antennas is placed; (**b**) Simulated normalized radiation pattern of two antennas when excited in opposite direction in case of target at freq. of 4 GHz.

Based on the above idea, we have implemented the proposed antenna in Robotics for pipeline blockage detection. Two antennas excited from opposite directions are placed at the open end of the C-arm of the robot, designed for pipeline as shown in Figure 19a. We placed one antenna at the upper arm and another one at the lower arm of the robot. Excitation current is applied at the upper arm, which is 180 degrees out of phase to that of the lower arm’s applied current. So, if there is any target between the two excited antennas at the same frequency, there will be a two counter scanning beams as shown in Figure 19b. This is the easiest way of detecting the target and it will pave the way for future research of scanning antennas for target detection in different industrial fields.

## 9. Conclusions

In this paper, we presented the bandwidth-enhanced and frequency scanning for developed fan beam array antenna utilizing novel technique of band-pass filter integration. First, a fan beam array antenna utilizing a grounded coplanar waveguide (GCPW) radiating element, CPW feed line, and the grounded reflector is developed for WiMAX applications. Secondly, a miniaturized wide-band bandpass filter is developed using SCSRR (Semi-Complementary Split Ring Resonator) and DGS (Defective Ground Structures) operating at the 3–8 GHz frequency band. Finally, the designed filter is integrated within the frequency scanning beam array antenna in a novel way to increase the impedance bandwidth as well as frequency scanning. The new frequency beam array antenna with integrated band-pass filter operates at 2.8 GHz to 6 GHz with a wide frequency scanning from the 50 to 125 ° range.

## Figures and Tables

**Table 1:** Comparison between proposed work and related work published in literature.

	Operating Freq.	Frequency Scanning	Technique Implemented
[9]	60 GHz	N/A	Reflector back array technique
[11]	4.65–10.5 GHz	N/A	GCPW Technique
[20]	1.8–1.95 GHz	N/A	RIS Technique
[23]	1.7–2.2 GHz	N/A	Non-parasitic grounded reflector Technique
This work	2.8–6 GHz	75 degree	Bandpass filter integration Technique with combination of GCPW, grounded reflector, and CPW feed line

**Table 2:** Achieved Results of the fan beam array antenna (utilizing reflector and without reflector).

Freq.		3.30 GHz		3.50 GHz	
		HFSS Results	Measured Results	HFSS Results	Measured Results
Relative SLL	Without reflector	−8.49 dB	-	−9.40 dB	-
	With reflector	−8.3 dB	−9.1 dB	−15.6 dB	−15.1 dB
Beam-width (3 dB)	Without reflector	30.0° × 99.0°	-	29.0° × 92.5°	-
	With reflector	28.0° × 90.0°	26° × 79.0°	28.5° × 89.5°	27.0° × 76.0°
Back-lobe-level (Max)	Without reflector	0 dB	-	0 dB	-
	With reflector	−9.50 dB	−12 dB	−13.95 dB	−14.4 dB

**Table 3:** Required values for the equivalent circuit model.

Type	Value	Frequency
*Z_om_*	50 Ω	-
*Z_sq_*	179 Ω	9.25 GHz
*Z_os_*	78.5 Ω	8.5 GHz
*Z_os_*	75.7 Ω	6.0 GHz
n	$\sqrt{Z_{om}/Z_{os}}$	-
